# Interferon Gamma-Inducible Protein 16 of Peripheral Blood Mononuclear Cells May Sense Hepatitis B Virus Infection and Regulate the Antiviral Immunity

**DOI:** 10.3389/fcimb.2021.790036

**Published:** 2021-11-18

**Authors:** Yu-Qing Lu, Jing Wu, Xiang-Ji Wu, Hui Ma, Yan-Xiu Ma, Rong Zhang, Meng-Nan Su, Nan Wu, Gong-Yin Chen, Hong-Song Chen, Xiao-Ben Pan

**Affiliations:** ^1^ Peking University People’s Hospital, Peking University Hepatology Institute, Beijing, China; ^2^ School of Basic Medical Sciences, Institute of Hepatology and Metabolic Diseases, Key Laboratory of Inflammation and Immunoregulation of Hangzhou, Key Laboratory of Aging and Cancer Biology of Zhejiang Province, Hangzhou Normal University, Hangzhou, China; ^3^ Department of Infectious Diseases & Department of Hepatology, Affiliated Hospital of Hangzhou Normal University, Hangzhou, China

**Keywords:** IFI16, HBV, PBMCs, innate immunity, interferon

## Abstract

Interferon gamma-inducible protein 16 (IFI16) is a DNA sensor protein, which triggers interferon-beta (IFN-β) production. However, the role of IFI16 in the innate immunity against hepatitis B virus (HBV) remains controversial. Peripheral blood mononuclear cells (PBMCs) and serum specimens were collected from 20 patients with chronic hepatitis B (CHB) receiving Peg-IFN-α2b therapy. IFI16 mRNA/protein of PBMCs and serum IFI16 at baseline and changes during Peg-IFN-α2b treatment were detected. The interaction between IFI16 and HBV DNA in the PBMCs was analyzed using chromatin immunoprecipitation assay. Leukemic T cell line CEM-C7 and HBV-replicating HepG2.2.15 cells were used to test the effects of interferon treatment and HBV replication on IFI16 expression. Compared with healthy controls, lower levels of *IFI16* mRNA but more significant expression of IFI16 protein with heterogeneous degradation were detected in PBMCs of CHB patients. Early changes in *IFI16* mRNA, but not *IFNB* mRNA of PBMCs or serum IFI16, were correlated to HBeAg seroconversion of Peg-IFN-α2b therapy. An interaction between IFI16 and HBV DNA was detected in the PBMCs. In the cultured HepG2.2.15 and CEM-C7 cells, interferons resulted in the translocalization of IFI16 from the cytoplasm to the nucleus and inhibited IFI16 degradation. IFI16 of PBMCs may play a role in sensing HBV infection, and early change in *IFI16* mRNA of PBMCs is valuable to predict HBeAg seroconversion in Peg-IFN-α2b treatment. The influences on IFI16 degradation and subcellular location may present a molecular mechanism of antiviral activity of interferon.

## Introduction

Hepatitis B virus (HBV) infections can be acute or chronic. The nature and strength of the host immune response determine the outcome of HBV infection (Bertoletti and Ferrari, 2012). It has been well established that adaptive immune responses by virus-specific CD4+ and CD8+ T cells, B cells, and antibodies are indispensable for HBV control. These responses lead to the elevation of alanine aminotransferase (ALT) and cytolytic and non-cytolytic elimination of the intracellular virus (Maini and Burton, 2019). Initiation of the innate immune response is essential for the induction of a sufficient level of antiviral adaptive immunity, in which recognition of conserved pathogen-associated molecular patterns (PAMPs) by cellular pattern recognition receptors (PRRs) is the first step. However, innate immune responses are predominantly weak or absent in the hepatocytes during the early phase of HBV infection (Dunn et al., 2009; Tsai et al., 2018). Previous studies have suggested that HBV may evade the innate sensing of PRRs and/or actively suppress the innate immune response in hepatocytes (Yu et al., 2010; Luangsay et al., 2015). However, the recent analyses of clinical samples showed that hepatocytes were lack of the downstream adaptor protein of stimulator of IFN genes (STING). HBV may bypass rather than interfere with the innate immune responses in hepatocytes (Dansako et al., 2016; Thomsen et al., 2016; Mutz et al., 2018; Suslov et al., 2018). These studies show that the initiation of the innate response in HBV infection remains unknown.

As a Pyrin and HIN domain (PYHIN) family member, interferon gamma-inducible protein 16 (IFI16) was recently identified as a PRR, which targets invading viral DNA genomes (Unterholzner et al., 2010; Dell’Oste et al., 2014). Upon sensing double-strand DNA (dsDNA) in the nucleus and cytoplasm, IFI16 stimulates the IRF-3 pathway through the STING to induce the expression of IFN-β or leads to the formation of the IFI16-ASC-Caspase-1 inflammasome complex and IL-1β production (Ansari et al., 2013; Thompson et al., 2014; Ansari et al., 2015). Recently, it was reported that IFI16 induces an IFN-β response during woodchuck hepatitis virus infection (Yan et al., 2016). In addition, intrahepatic *IFI16* mRNA level is associated with HBV clearance and closely related to the degree of inflammation of liver tissues in a patient with chronic hepatitis B (CHB) (Wieland et al., 2004; Pang et al., 2018; Yang et al., 2020). However, this protein has been detected in intrahepatic Kupffer cells, endothelial cells, NK cells, dendritic cells, and hepatic stellate cells except for hepatocytes in the liver (Pang et al., 2018). In this context, it remains to clarify whether IFI16 can sense HBV infection and which type of cells expressing IFI16 is involved in the antiviral immunity.

The intrahepatic and circulating immunocyte populations play an essential role in the antiviral immunity during HBV infection. Although these immunocytes cannot support HBV infection and replication, previous studies demonstrated that HBV DNA is detectable in the immunocytes at every stage of chronic HBV infection (Pasquinelli et al., 1986; Vicenti et al., 2018). As a cell population for presenting epitope, immunocytes are powerful to degrade viral proteins. It indicates that the PRRs of immunocytes stand a chance to sense HBV DNA engulfment and regulate antiviral immunity. In the present study, using clinical specimens from CHB patients, we detected changes of IFI16 expression in peripheral blood mononuclear cells (PBMCs) and serum and analyzed its correlation with HBeAg seroconversion in Peg-IFN-α2b therapy. In addition, we detected the interaction effects among the IFI16 expression, HBV replication, and interferon treatment. Our study may help to assess the role of IFI16 in anti-HBV innate immunity and its clinical significance.

## Materials and Methods

### Cohorts of Patients With Chronic HBV Infection

Specimens in this study were collected from the CHB patients who were previously recruited for clinical studies of Peg-IFN-α2b (Schering-Plough, Kenilworth, NJ, USA) therapy at Peking University People’s Hospital. The criteria for the patients’ screening were described previously (Pan et al., 2012). The patients were injected with Peg-IFN-α2b at a dose of 1 to 1.5 mg/kg per week for 24 to 48 weeks. The efficacy was assessed at the end of the treatment and after 24 weeks of follow-up. Control PBMC samples were collected from healthy donors without clinical complaints and HBV infection at the time of donation.

The PBMCs were isolated by standard density gradient centrifugation using Ficoll-Paque (Amersham Biosciences, Freiburg, Germany). The PBMC specimens were stored in liquid nitrogen, and sera were stored at -80°C until further analysis. A total of 20 matched sera samples and RNA samples of PBMCs at baseline and 2 weeks after Peg-IFN-α2b therapy were used for analysis. Among them, a total of 12 protein samples of PBMCs from baseline patients were available for analysis.

### Cell Cultures

Hepatic cell line HepG2 was maintained in complete DMEM (Invitrogen, Carlsbad, CA, USA). HepG2.2.15 cell lines, which support a stable HBV replication, were maintained in complete DMEM supplemented with 380 μg/mL of G418 antibiotic (Sigma-Aldrich, St. Louis, MO, USA). The human leukemic T cell line CEM-C7 was maintained in a complete RPMI-1640 medium (Invitrogen).

### RNA Isolation and Quantification

Total RNA from PBMCs was isolated using a Trizol kit (Invitrogen). One microgram of total RNA was used for one-step quantitative reverse transcription PCR (RT-qPCR) with SYBR green detection system (NEB, Ipswich, Massachusetts, USA). The β-actin gene was used as a control to normalize for variations. Primers for amplifying target genes were synthesized (SBS Bio, Beijing, China) and are shown in **Table S1**.

### Western Blot

Cells were lysed with 1×Laemmli buffer, and a fraction of the cell lysate was separated on 10% polyacrylamide gels and transferred to polyvinylidene difluoride membranes. The membranes were probed with primary antibodies for IFI16 (Abcam, Chicago, IL, USA), followed by incubation with secondary antibodies conjugated with horseradish peroxidase (anti-msIgG-HRP, Abcam), housekeeping protein β-actin was used as a control. Signals were detected using chemiluminescence. The images were obtained and analyzed using QuantityOne software (Bio-Rad, Hercules, CA, USA).

### Chromatin Immunoprecipitation Assay

According to the manufacturer’s guidelines, the ChIP assay was performed using a Pierce Agarose ChIP kit (Invitrogen). Briefly, 2 × 10^6^ cells were fixed in 1% formaldehyde and 1 × glycine solution. The cells were pelleted by centrifugation and resuspended in immunoprecipitation lysis buffer supplemented with a proteinase inhibitor cocktail. Fifty microliters of the cell lysates were incubated with 4 μL of mouse anti-IFI16; anti-RNA polymerase II antibody and normal rabbit IgG were used as positive and negative controls, respectively. ChIP Grade Protein A/G Plus Agarose (Sigma-Aldrich) was used for target-specific IP. The obtained DNA was then subjected to PCR analysis for detection of HBV DNA. The primers used are listed in **Table S1**.

### Enzyme-Linked Immunosorbent Assays and HBV DNA Quantification

According to the manufacturer’s instructions, serum IFI16 was detected using an ELISA kit (Sangong, Shanghai, China). HBV serological markers were detected by a chemiluminescence immunoassay (Abbott Laboratories, Abbott Park, IL, USA) on an automatic immunoassay analyzer (ARCHITECT i2000, Abbott), and HBV DNA was quantified using a qPCR kit on a LightCycler instrument (Cobas Taqman; Roche, Mannheim, Germany).

### Immunofluorescent Staining and Confocal Analysis

Cell cultures in 8-well chambers were fixed with 4% paraformaldehyde and permeabilized by incubation with 0.1% Triton X-100. Cells were probed with primary antibodies specific for IFI16 (Abcam), and bound primary antibodies were visualized by Alexa Fluor 488-conjugated goat anti-mouse IgG or 594-conjugated goat anti-rabbit IgG (Invitrogen). Cell nuclei were stained with Hoechst33342 (Invitrogen), and images were acquired by a confocal microscope (TCS-NT, Leica, Germany).

### Statistical Analysis

Statistical analysis was performed using SPSS software (V.16.0, SPSS Inc, Chicago, IL, USA). Based on the data type, normality, and variance features, the paired Student’s *t*-test or Mann-Whitney U test were selected to evaluate differences. Pearson’s correlation analysis was used to evaluate correlations. *P* < 0.05 was considered statistically significant.

### Ethics Issues

This study was conducted following the ethical guidelines of the Declaration of Helsinki and was approved by the Ethics Committee of Peking University People’s Hospital. Due to the use of residual specimens collected ten years ago, the requirement to obtain informed patient consent was waived by the Ethics Committee.

## Results

### Characteristics of CHB Patients Receiving Peg-IFN-α2b Treatment

The cohort of CHB patients included 16 males and 4 females. The average age was 30 years. The ALT level at baseline was 119 U/L, and it decreased to 38 U/L by the end of treatment. The median level of HBV DNA was decreased by 2.94 log10 after 24 weeks of Peg-IFN-α2b treatment. Eight patients achieved HBeAg seroconversion at the end of treatment. However, the reconversion of HBeAg occurred in one patient during the 24-week follow-up (**Table 1**).

**Table 1:** Characteristics of patients with chronic HBV infection.

Characteristics	Baseline	Week 4	EOT	F24w
Number (Male: Female)	20 (16: 4)
Age (years), mean ± SD	30.08 ± 6.74
ALT (U/L), median (range)	119.50 (32–405)	100.50 (29–475)	38.00 (17–93)	42.00 (8–371)
AST (U/L), median (range)	70.00 (25–586)	59.50 (33–288)	34.00 (21–75)	30.00 (13–255)
HBV DNA (log_10_ IU/mL), median (range)	8.30 (5.21–9.98)	6.25 (4.18–8.41)	5.36 (2.51–8.55)	4.98 (2.58–9.56)
HBsAg (IU/mL), median (range)	5675.97 (316–59549)	4199.90 (82–561773)	1885.43 (10–26461)	3101.94 (3.3–41025)
HBeAg (IU/mL), median(range)	213.02 (2.59–2067.33)	40.72 (0.25–1737.05)	16.22 (0.84–784.04)	92.88 (0.23–2120.72)
HBeAg seroconversion (n)	NA	0	8	7

EOT, end of treatment.

F24w, follow-up for 24 weeks.

### HBV DNA Interacts With IFI16 and Inhibits IFI16 Degradation in the PBMCs of Patients With CHB

**Figure 1:**
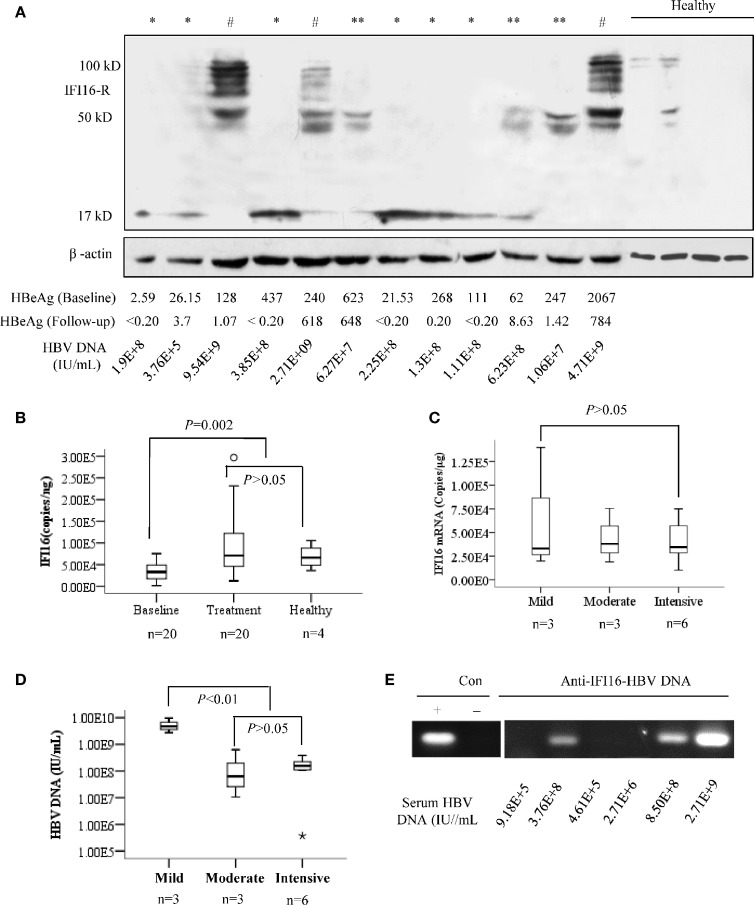
IFI16 expression and degradation in the peripheral blood mononuclear cells (PBMCs) of chronic hepatitis B (CHB) patients. PBMCs from 12 patients with CHB and four healthy donors were included for analysis. Based on the fragment migration in the PAGE gel, IFI16 degradation in PBMCs was classified into three patterns, including mild degradation (#, full length IFI16 at 95-100 kDa with fragments at 40-90 kDa), moderate degradation (**, 40-50 kDa), and intensive degradation (*, ~17 kDa). β-actin was used as an internal control for Western blot **(A)**. Mann-Whitney U test evaluated differences in *IFI16* mRNA levels of PBMCs of patients at baseline, after two weeks of Peg-IFN-α2b treatment, and healthy donors **(B)**. Mann-Whitney U test evaluated differences in IFI16 mRNA of PBMCs with different IFI16 degradation **(C)**. Serum HBV DNA levels were classified into three groups according to the degree of IFI16 degradation. Mann-Whitney U test was used to evaluate differences in HBV DNA levels among the groups **(D)**. ChIP assay was used for analyzing the interaction between IFI16 and HBV DNA in PBMCs of the baseline patients with CHB. The obtained DNA was subjected to PCR analysis of HBV DNA **(E)**.

The *IFI16* mRNA levels of PBMCs from healthy individuals were significantly higher than those of CHB patients at baseline (*P*=0.002), while two weeks of Peg-IFN-α2b treatment rescued the *IFI16* mRNA levels in CHB patients. On the contrary, negative or only trace IFI16 expression was detected in PBMCs from healthy individuals, while a significant expression with heterogeneous degradation of IFI16 protein was detected in the PMBCs of CHB patients. Among them, intensive IFI16 degradation with fragments at sizes of ~17 kDa was detected in six patients; moderate IFI16 degradation with fragments at sizes of ~40 and 50 kDa was detected in three patients, and strong IFI16 expression with mild degradation (presence of full-length IFI16) was detected in three patients (**Figures 1A, B**). There was no significant difference in *IFI16* mRNA levels among the PBMCs with different protein degradation (**Figure 1C**). Patients with mild degradation of IFI16 in PBMCs had a significantly higher load of serum HBV DNA than patients with moderate or intensive IFI16 degradation (**Figure 1D**, *P <*0.01). In addition, a significant interaction between HBV DNA and IFI16 was detected in the PBMCs of patients with high loads of serum HBV DNA (**Figure 1E**).

### Early Changes in *IFI16* mRNA but Not *IFNB* mRNA of PBMCs Were Associated With HBeAg Seroconversion in Peg-IFN-α2b Therapy

**Figure 2:**
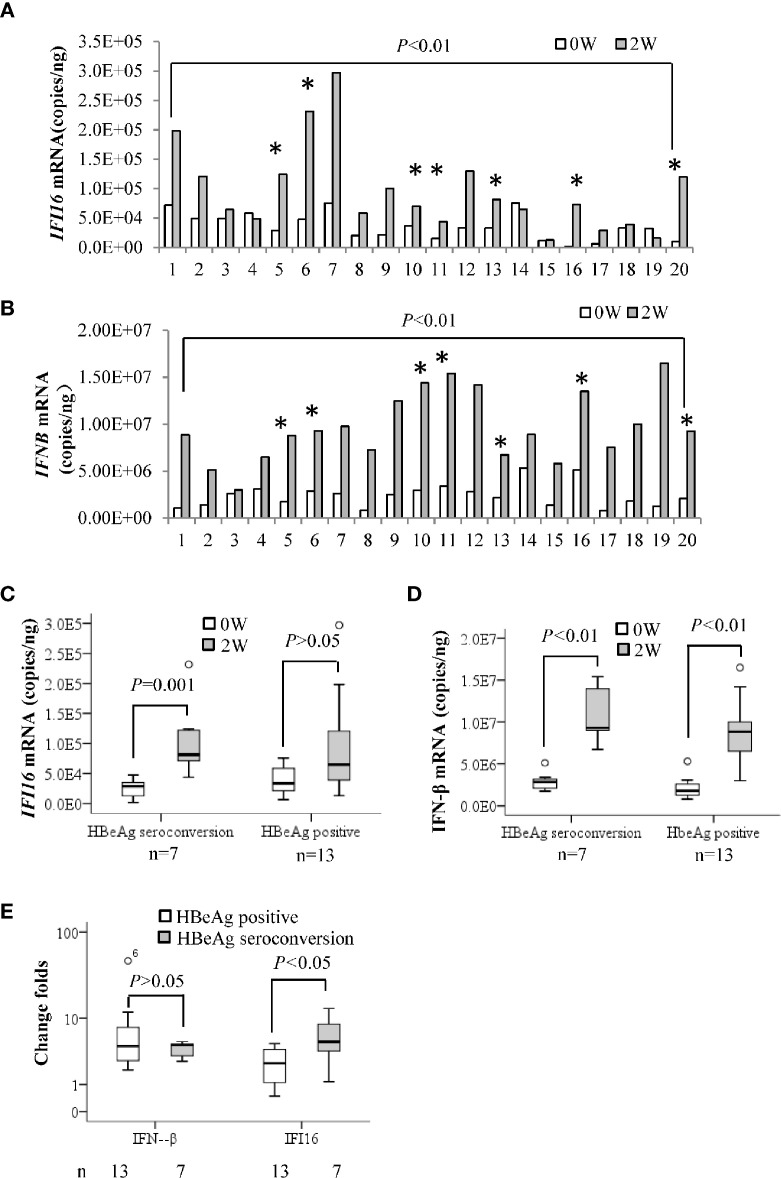
Early changes in *IFI16* mRNA of PBMCs in patients with CHB treated with interferon. Quantification of *IFI16* mRNA **(A)** and *IFN-β* mRNA **(B)** in peripheral blood mononuclear cells (PBMCs) at baseline and two weeks after Peg-IFN-α2b treatment were comparatively analyzed by a paired Student’s t-test. * Indicates patients achieving HBeAg seroconversion at 24 weeks follow-up after Peg-IFN-α2b therapy. The IFI16 mRNA **(C)** and IFN-β mRNA **(D)** changes in the patients with or without HBeAg seroconversion were tested by performing a Mann-Whitney U test. The patients were grouped according to HBeAg seroconversion. The difference in change folds of *IFI16* mRNA and *IFN-β* mRNA after 2 weeks of interferon treatment were compared using a Mann-Whitney U test **(E)**. *P* < 0.05 was considered statistically significant.

*IFI16* and *IFN-β* mRNA levels in PBMCs at baseline and 2 weeks after Peg-IFN-α2b treatment were grouped according to HBeAg seroconversion. Both *IFI16* and *IFN-β* mRNA levels in PBMCs were significantly increased after 2 weeks of Peg-IFN-α2b treatment (*P* < 0.01, **Figures 2A, B**). The median value of *IFI16* mRNA levels increased by 5-fold in seven patients who achieved HBeAg seroconversion (*P* = 0.001). However, the change was insignificant in patients without HBeAg seroconversion (*P* > 0.05, **Figure 2C**). The median values of *IFN-β* mRNA increased by 5.0- and 4.5-fold in patients with or without HBeAg seroconversion, respectively (*P* < 0.01. **Figure 2D**), and there was no significant difference in the change folds of *IFN-β* mRNA between the two groups (*P* > 0.05. **Figure 2E**).

### Serum IFI16 Levels Did Not Significantly Change in Peg-IFN-α2b Treatment

**Figure 3:**
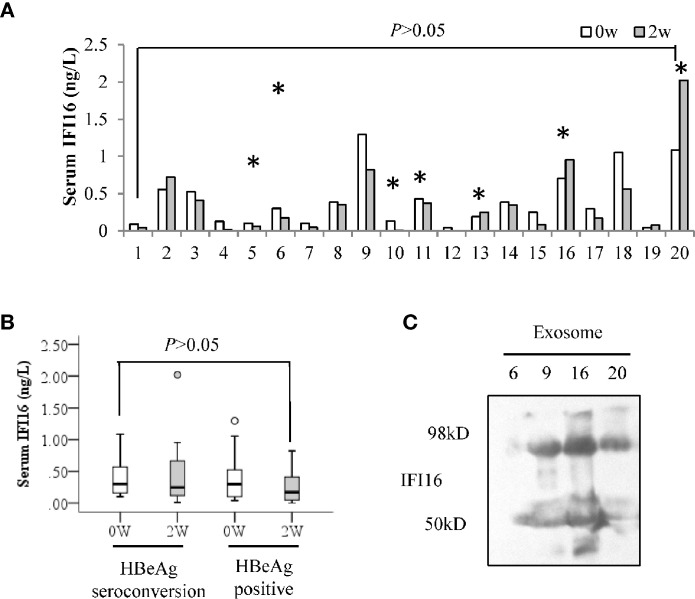
Serum IFI16 levels in the CHB patients treated with interferon. Serum IFI16 levels at baseline and 2 weeks after Peg-IFN-α2b treatment were comparatively analyzed by a paired Student’s *t*-test. * patients achieved HBeAg seroconversion at 24 weeks follow-up after Peg-IFN-α2b therapy **(A)**. Patients were grouped according to the HBeAg seroconversion, and the changes of serum IFI16 after 2 weeks of Peg-IFN-α2b treatment were tested by performing a Mann-Whitney U test **(B)**. IFI16 expression of sera exosomes from the CHB patients of baseline. A total of 500 μL of sera from four CHB patients was used to extract 100 μL of total exosomes. IFI16 expression of exosomes was detected using Western blot **(C)**.

ELISA analysis showed that serum IFI16 levels did not significantly change in the CHB patients after 2 weeks of Peg-IFN-α2b treatment (**Figure 3A**). In addition, there was no significant difference in the serum IFI16 levels even when these patients were grouped according to HBeAg seroconversion, regardless of at baseline or 2 weeks after Peg-IFN-α2b treatment (**Figure 3B**). Western blotting detected both IFI16 and degraded fragments in the purified serum exosomes from the CHB patients (**Figure 3C**).

### Interferon Promoted Nuclear Expression and Inhibited Degradation of IFI16

**Figure 4:**
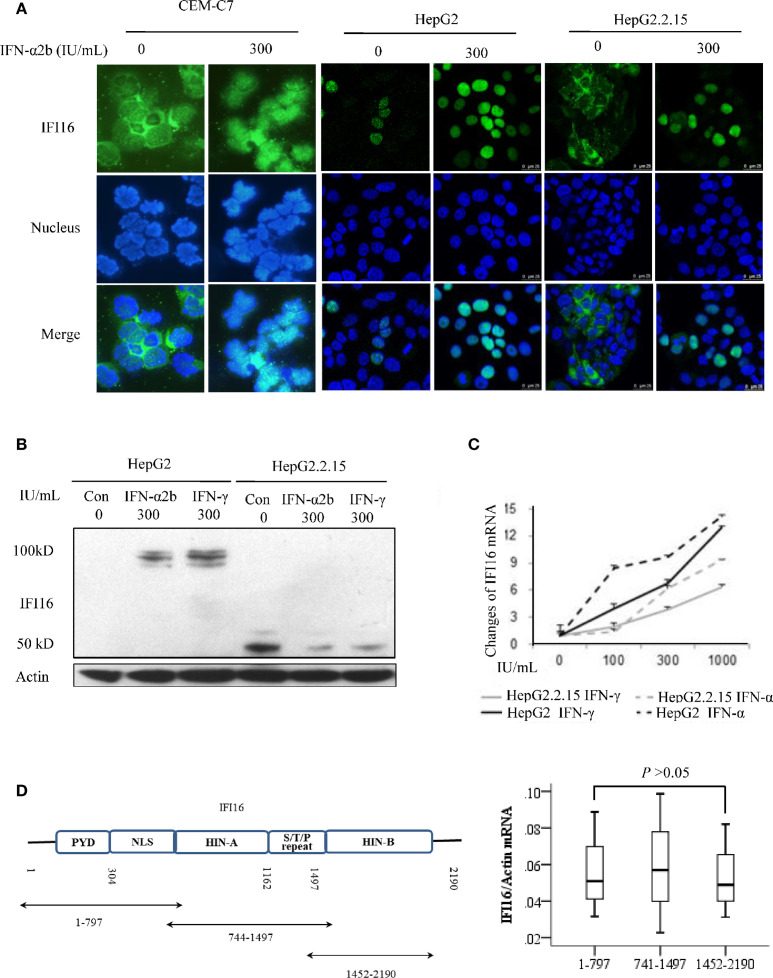
Interferon enhances expression, promotes translocation, and inhibits degradation of IFI16. Immunofluorescent staining for detecting the subcellular distribution of IFI16 in the CEM-C7, HepG2, and HepG2.2.15 cells after 2 days of IFN-α2b treatment. DAPI was used for indicating nucleus **(A)**. IFI16 protein **(B)** and *IFI16* mRNA **(C)** in HepG2 and Hep2.2.15 cells after 2 days of IFN-α2b or IFN-γ treatment. The RT-qPCR analysis of overlapping segments of *IFI16* mRNA in HepG2.2.15 cells. The difference was tested by performing a Mann-Whitney U test **(D)**.

The immunofluorescent staining showed that IFI16 was detected in the cytoplasm of human leukemic T cell line CEM-C7, while the protein transferred from the cytoplasm to the nucleus after two days of 300 IU/mL of IFN-α2b treatment. IFI16 was expressed in the nucleus of 20% of parent HepG2 cells, and the IFN-α2b treatment significantly promoted its expression. In the HBV-replicating HepG2.2.15 cells, IFI16 is primarily expressed in the cytoplasm, and the protein is transferred into nucleic after IFN-α2b treatment (**Figure 4A**). Western blot analysis showed that the full-length of IFI16 was detected in the HepG2 cells treated with interferons. However, only IFI16 fragments at sizes of ~40 and 50 kDa were detected in HepG2.2.15 cells, and IFN-α2b or IFN-γ treatment reduced their expression (**Figure 4B**). In addition, compared with that of HepG2 cells, the IFNs-inducible transcription of *IFI16* mRNA was markedly reduced in the HepG2.2.15 cells (**Figure 4C**). To verify whether the IFI16 fragments were produced at the mRNA level, three overlapping segments of *IFI16* mRNA were quantitatively amplified. The RT-qPCR showed that there was no significant difference in the mRNA quantifications among these segments (**Figure 4D**), suggesting that IFI16 fragments were produced at the post-translational level.

## Discussion

In the present study, based on the analyses of clinical specimens from patients with chronic HBV infection, we found that IFI16 is largely degraded in the PBMCs, and the interaction with HBV DNA reduces the degradation. In addition, interferon treatment inhibits IFI16 degradation and results in its translocation from cytoplasm to nucleus. Further, the early changes in *IFI16* mRNA of PBMCs are predictive of HBeAg seroconversion in the CHB patients receiving Peg-IFN-α2b treatment therapy.

According to therapeutical response at the 24-week follow-up (Tab. 1), we divided the patients into groups of baseline, HBeAg seroconversion, and HBeAg positive (non-seroconversion). The previous studies showed that expression of PRRs and their intracellular signaling were inhibited in chronic HBV infection (Wu et al., 2009; Yu et al., 2010; Luangsay et al., 2015; Tsai et al., 2018). Consistently, reduced transcription of *IFI16* mRNA was detected in the PBMCs of baseline CHB patients, while Peg-IFN-α2b therapy resecured the transcriptional level (**Figure 1B**). On the contrary, IFI16 protein was barely detected in the PBMCs and blood smears of healthy individuals. However, a remarkable cytoplasmic expression of IFI16 and/or degraded fragments were detected in the patients with CHB, particularly those with high loads of serum HBV DNA. In addition, an interaction between IFI16 and HBV DNA was detected in the PBMCs (**Figure 1** and **Figure S1**). These results showed that IFI16 of PBMCs can sense HBV DNA of engulfment, and sufficient interaction between HBV DNA and IFI16 may inhibit the degradation of IFI16. However, it might be noticed that the possible interaction between engulfed HBV capsid and IFI16 may also result in positive HBV DNA detection. In addition, due to the significantly different IFI16 expression detected *in vivo* and *in vitro* (**Figure S1**), we suggest that IFI16 expression in cultured PBMCs may not reflect the real expression of IFI16 *in vivo*.

The previous study demonstrated that the *IFI16* mRNA of liver tissues is correlated with the HBeAg seroconversion and supposed that IFI16 may directly inhibit transcription of HBV cccDNA (Yang et al., 2020). However, it is paradoxical because IFI16 was detected in the intrahepatic immunocytes but not hepatocytes in the liver of CHB patients (Similar results were detected in our study, **Figure S2**) (Pang et al., 2018). In the present study, we showed that the *IFI16* mRNA of PBMCs increased after interferon treatment, and the early changes were correlated to the achievement of HBeAg seroconversion (**Figures 2A, C**). In this context, it is supposed that the immunocyte populations might be the primary source of the *IFI16* mRNA of liver tissues, which may explain the previous paradox regarding intrahepatic *IFI16* expression and HBeAg seroconversion (Pang et al., 2018; Yang et al., 2020). Of interest, the change of *IFNB* mRNA levels was not correlated with HBeAg seroconversion in interferon treatment (**Figures 2B, D**). Compared with IFN-β, IFI16 is more specifically linked to the innate immunity against DNA virus, which may support the better correlation between IFI16 expression and HBeAg seroconversion.

Exosome has been demonstrated to make a significant contribution to immune responses (Robbins and Morelli, 2014; Robbins et al., 2016). In this study, IFI16, and the fragments were also detectable in the serum exosomes (**Figure 3C**), suggesting a potential way of IFI16-mediated immune regulation. However, serum IFI16 levels did not significantly change in the Peg-IFN-α2b treatment (**Figures 3A, B**). The analysis of IFI16 expression in the cultured CEM-C7 and HepG2.2.15 cells showed that interferon treatment resulted in translocation of IFI16 from the cytoplasm to the nucleus (**Figure 4A**). Since cytoplasmic location is the first step of IFI16 excretion, it is logical that the translocation of IFI16 will impair its excretion, thereby affecting the serum IFI16 concentration. In addition, it is noteworthy whether interferon therapy will also change the IFI16 expression and forms in exosomes, which may amplify the effect of immune regulation by interferon.

Similar to detecting IFI16 fragments in PBMCs, the typical IFI16 fragments of ~40-50 kDa were also detected in HepG2.2.15 cells. Theoretically, these fragments could be produced from mRNA splicing or protein degradation. However, the RT-qPCR analysis showed no significant difference in the number of overlapping segments of *IFI16* mRNA (**Figure 4D**), indicating that the IFI16 fragments should not be produced at the mRNA level. Interferons, including IFN-α and IFN-γ, can significantly inhibit IFI16 degradation, which may indicate a novel mechanism of interferon-mediate regulation of IFI16 expression, in addition to promoting its transcription (**Figure 4D**). However, compared with that in HepG2 cells, interferon-inducible transcription of IFI16 was significantly impaired in HepG2.2.15, which may explain the barely detected full-length IFI16 in the HepG2.2.15 cells (**Figures 4B, C**).

Interestingly, the rule of IFI16 degradation seems to be inconsistent because IFI16 degradation was inhibited in the PBMCs of CHB patients but was promoted in HepG2.2.15 cells. We deduce that the distinct subcellular distribution of IFI16 may cause the difference in degradation. IFI16 is expressed in the naive HepG2 cells, while the interaction between IFI16 and HBV cccDNA occurred in the nuclei of HepG2.2.15, leading to the translocation of IFI16 from the nuclei to the cytoplasm in which the IFI16 cleavage occurred. However, IFI16 is expressed in the cytoplasm and is physiologically degraded in the immunocytes. In this case, the interaction between IFI16 and HBV DNA hampers the cleavage and accumulates IFI16 in the cytoplasm.

In conclusion, despite the limited number of clinical specimens, we demonstrated that IFI16 of PBMCs may sense HBV DNA during the infection. The early changes in IFI16 mRNA are valuable to predict HBeAg seroconversion in interferon treatment. In addition, interferon can inhibit the IFI16 degradation and promote its nuclear relocation. These findings may help elucidate innate immunity mechanisms against HBV infection and the regulation of IFI16 by interferon activity.

## Data Availability Statement

The original contributions presented in the study are included in the article/**Supplementary Material**. Further inquiries can be directed to the corresponding authors.

## Ethics Statement

The studies involving human participants were reviewed and approved by Ethics Committee of Peking University People’s Hospital. The ethics committee waived the requirement of written informed consent for participation.

## Author Contributions

Y-QL, JW, X-JW, Y-XM, and HM are co-first authors. X-BP and H-SC designed the study and interpreted the results. X-BP drafted the manuscript, and NW revised the manuscript. Y-QL, JW, Y-XM, X-JW, RZ, and M-NS completed the experiments and data analysis. HM and G-YC provided the clinical specimens. All authors contributed to the article and approved the submitted version.

## Funding

This study was supported by Grants from the National Natural Science Foundation of China (No. 82070610 and 81670530), and the Start-up Foundation of Hangzhou Normal University No. 2018QDL035).

## Conflict of Interest

The authors declare that the research was conducted in the absence of any commercial or financial relationships that could be construed as a potential conflict of interest.

## Publisher’s Note

All claims expressed in this article are solely those of the authors and do not necessarily represent those of their affiliated organizations, or those of the publisher, the editors and the reviewers. Any product that may be evaluated in this article, or claim that may be made by its manufacturer, is not guaranteed or endorsed by the publisher.

## Supplementary Material

Supplementary Figure 1 The subcellular distribution of IFI16 in immunocytes. IFI16 was barely detected in the immunocytes, while it was significantly expressed in the cytoplasm of immunocytes in some CHB patients **(A)**. In the PBMCs after two days of culture, IFI16 was significantly expressed in the nuclei and occasionally in the cytoplasm of healthy PBMCs (white arrows), while IFN-α2b treatment promotes its nuclear expression **(B)**.

Supplementary Figure 2 The expression of IFI16 in liver tissues of patients with chronic HBV infection. CK18 was used to indicate the marker of the hepatocyte (green arrow). As shown in the representative figures, IFI16 was negative in the hepatocytes, while aggregated **(A)** or scattered **(B)** IFI16-positive cells were detected in the inflammatory areas in the liver tissues of CHB patients. The white arrows indicated the inflammatory areas with aggregation of inflammatory cells.
