# Supplementary figures and images for: Interferon Gamma-Inducible Protein 16 of Peripheral Blood Mononuclear Cells May Sense Hepatitis B Virus Infection and Regulate the Antiviral Immunity
Source: Front Cell Infect Microbiol. 2021 Nov 18;11:790036. doi: 10.3389/fcimb.2021.790036 (PMC8637547; doi:10.3389/fcimb.2021.790036)

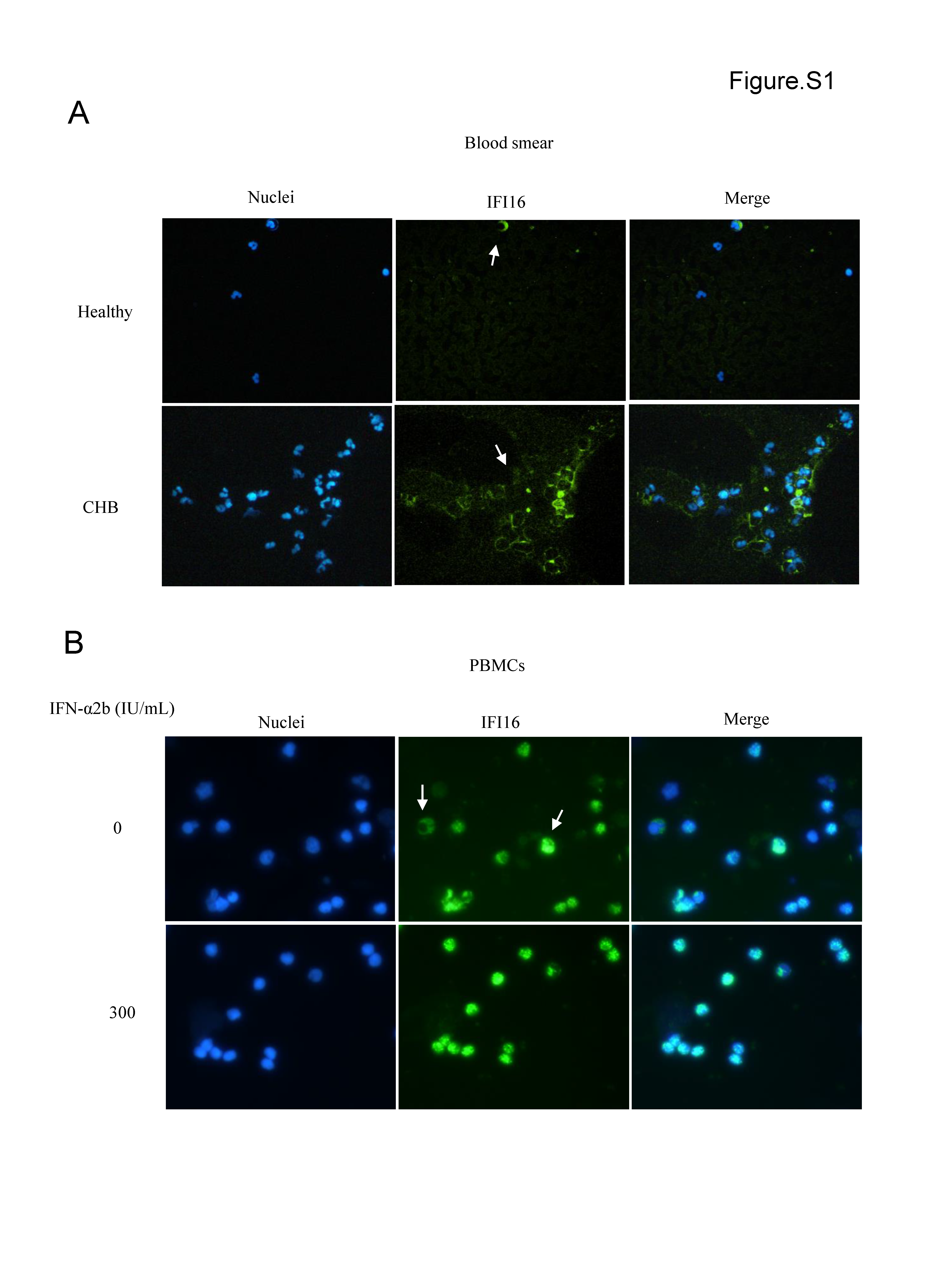

Supplement: Supplementary Figure 1 — The subcellular distribution of IFI16 in immunocytes. IFI16 was barely detected in the immunocytes, while it was significantly expressed in the cytoplasm of immunocytes in some CHB patients (A). In the PBMCs after two days of culture, IFI16 was significantly expressed in the nuclei and occasionally in the cytoplasm of healthy PBMCs (white arrows), while IFN-α2b treatment promotes its nuclear expression (B). [file Image_1.tiff]

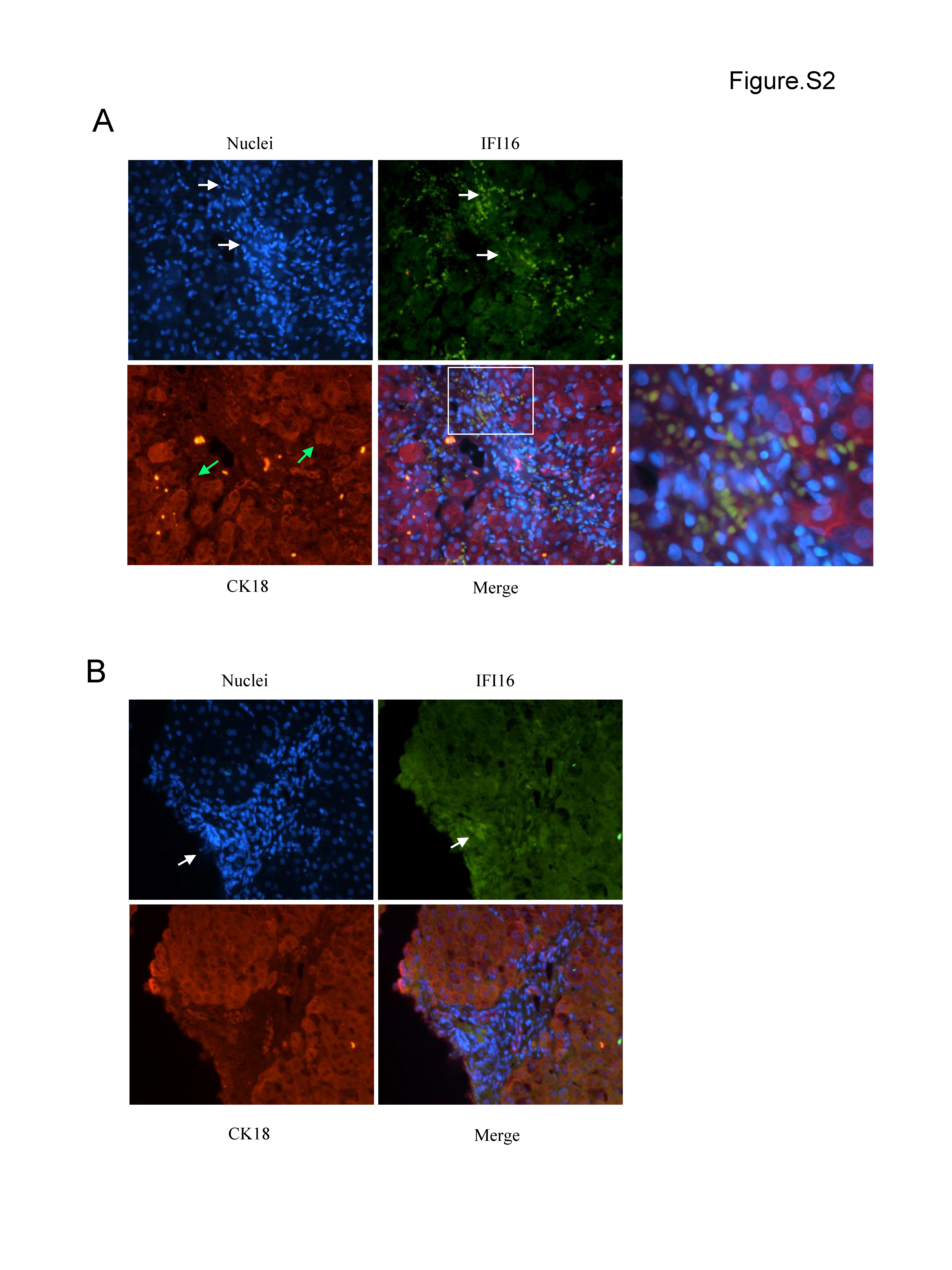

Supplement: Supplementary Figure 2 — The expression of IFI16 in liver tissues of patients with chronic HBV infection. CK18 was used to indicate the marker of the hepatocyte (green arrow). As shown in the representative figures, IFI16 was negative in the hepatocytes, while aggregated (A) or scattered (B) IFI16-positive cells were detected in the inflammatory areas in the liver tissues of CHB patients. The white arrows indicated the inflammatory areas with aggregation of inflammatory cells. [file Image_2.tiff]
