# Supplementary material for: Interferon Gamma-Inducible Protein 16 of Peripheral Blood Mononuclear Cells May Sense Hepatitis B Virus Infection and Regulate the Antiviral Immunity
Source: Front Cell Infect Microbiol. 2021 Nov 18;11:790036. doi: 10.3389/fcimb.2021.790036 (PMC8637547; doi:10.3389/fcimb.2021.790036)
Supplement: Supplementary file 3 [file Table_1.docx]

**Table S1: Sequences of the primers for PCR**

| Gene | Sequence (5′ to 3′) | Notes |
| --- | --- | --- |
| IFI16 531-853 | F: CAACAGTTCTTCAACTGAGAAC  R: TAGGACCAGCTTCAGATACAG | qPCR |
| IFI16 1-797 | F: ATGGGAAAAAAATACAAGAAC  R: TTCCAAATAATCTGATATGATG | qPCR |
| IFI16 744-1497 | F: TGAAGGAGAAATTCAATGGAAAG  R: ACGTGGTTAAGAAACTGCTGCT | qPCR |
| IFI16 1452-2190 | F: CCTCAGATGCCTCCATCAACACC  R: TTAGAAGAAAAAGTCTGGTGAAG | qPCR |
| IFN-β | F: AAACTCATGAGCAGTCTGCA | qPCR |
|  | R: AGGAGATCTTCAGTTTCGGAGG |  |
| β-actin | F: GCGGGAAATCGTGCGTGACATT | qPCR |
|  | R: GATGGAGTTGAAGGTAGTTTCGTG |  |
| HBV1377-1628 | F: GCTGCTAGGCTGTGCTGC | For ChIP |
|  | R: CGTTCACGGTGGTCTCCATG. |  |
